# Supplementary figures and images for: Determining the numbers of a landscape architect species (Tapirus terrestris), using footprints
Source: PeerJ. 2018 Mar 29;6:e4591. doi: 10.7717/peerj.4591 (PMC5878928; doi:10.7717/peerj.4591)

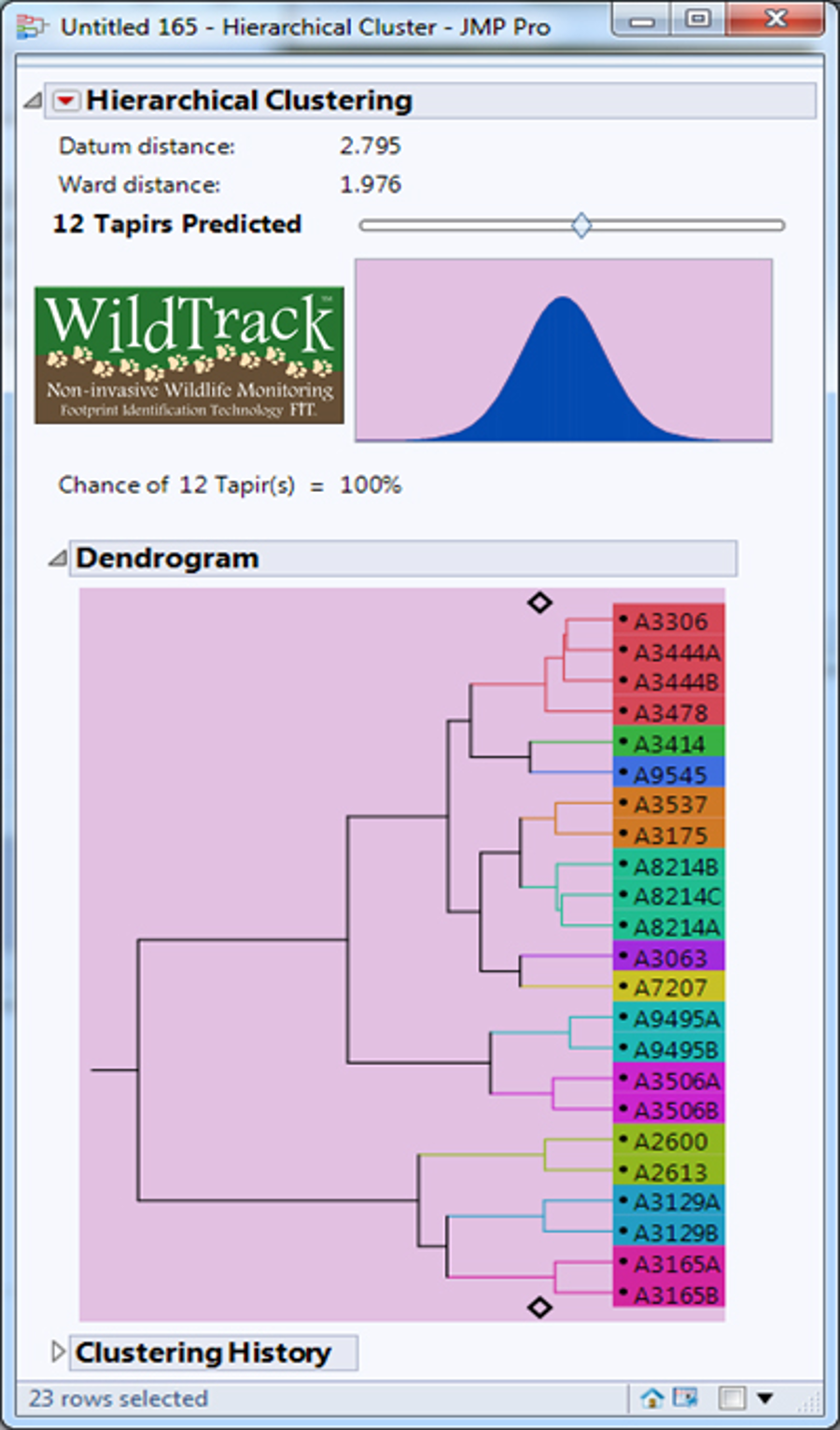

Supplement: Supplemental Information 1 — Each letter represents a location; a number represents a trail; a number and letter, a sub-trail; and the colors represent one individual. [file peerj-06-4591-s001.png]

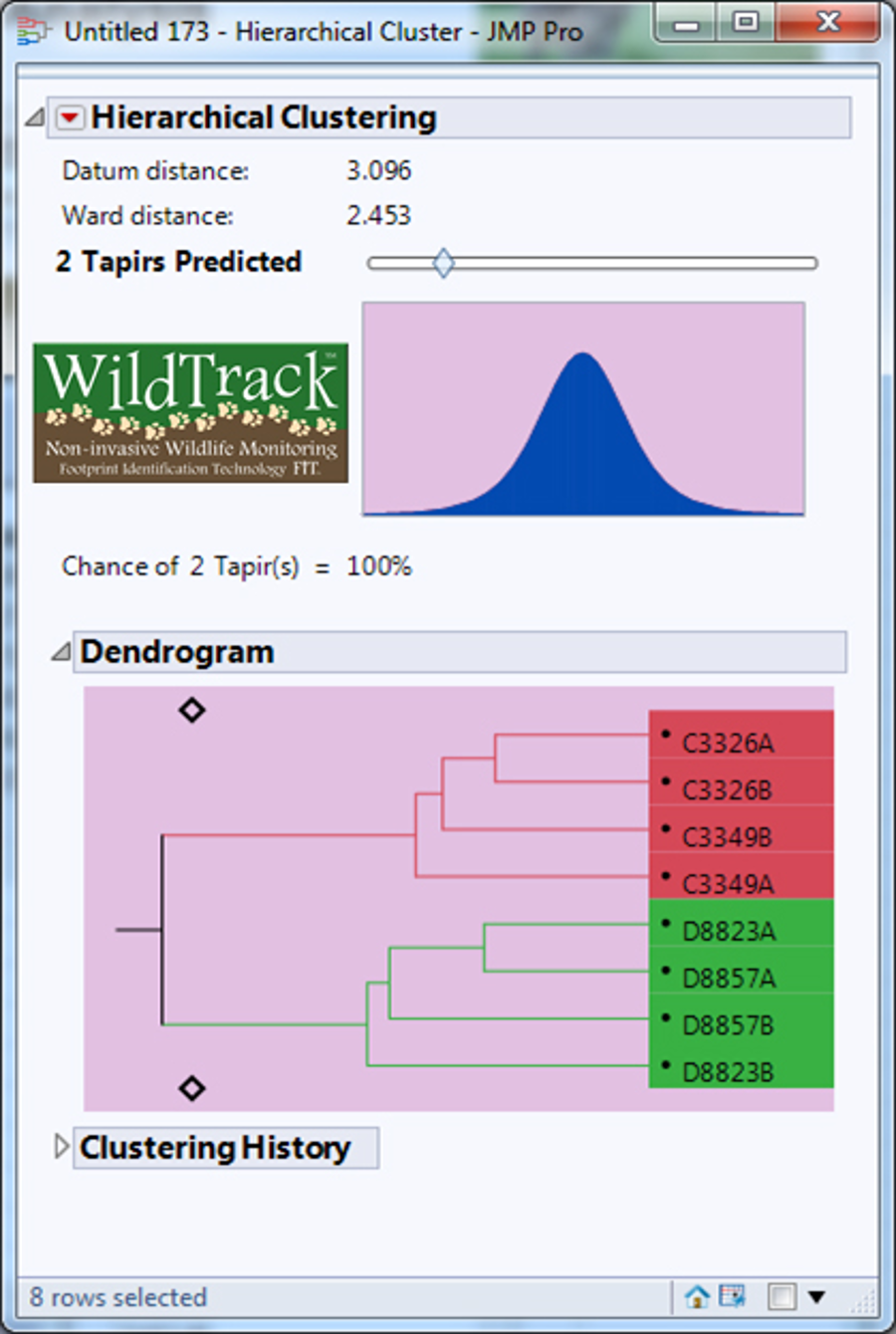

Supplement: Supplemental Information 2 — Each letter represents a location; a number represents a trail; a number and letter, a sub-trail, and the colors represent one individual. [file peerj-06-4591-s002.png]

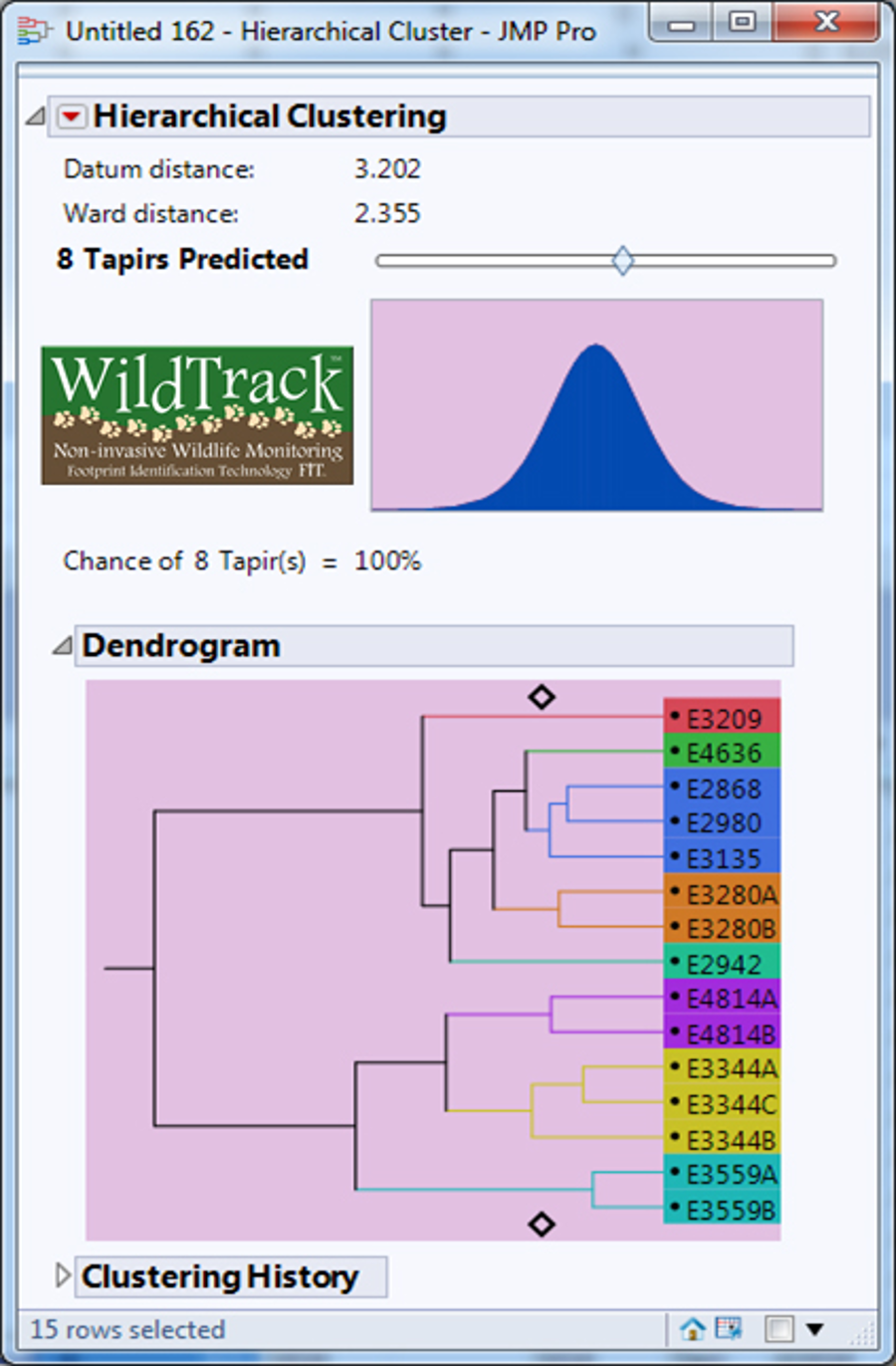

Supplement: Supplemental Information 3 — Each letter represents a location; a number represents a trail; a number and letter, a sub-trail, and the colors represent one individual. [file peerj-06-4591-s003.png]

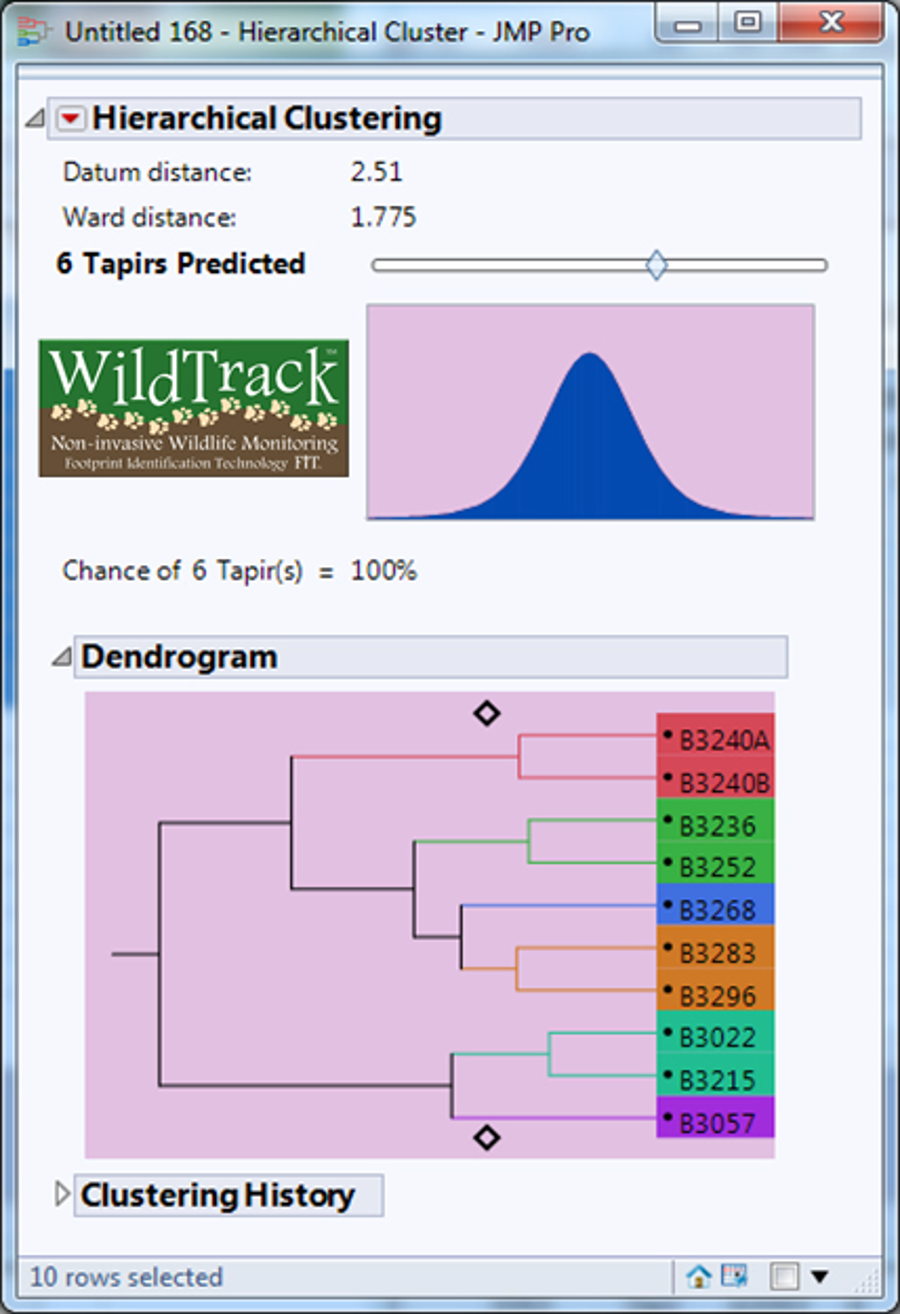

Supplement: Supplemental Information 4 — Each letter represents a location; a number represents a trail; a number and letter, a sub-trail, and the colors represent one individual. [file peerj-06-4591-s004.png]

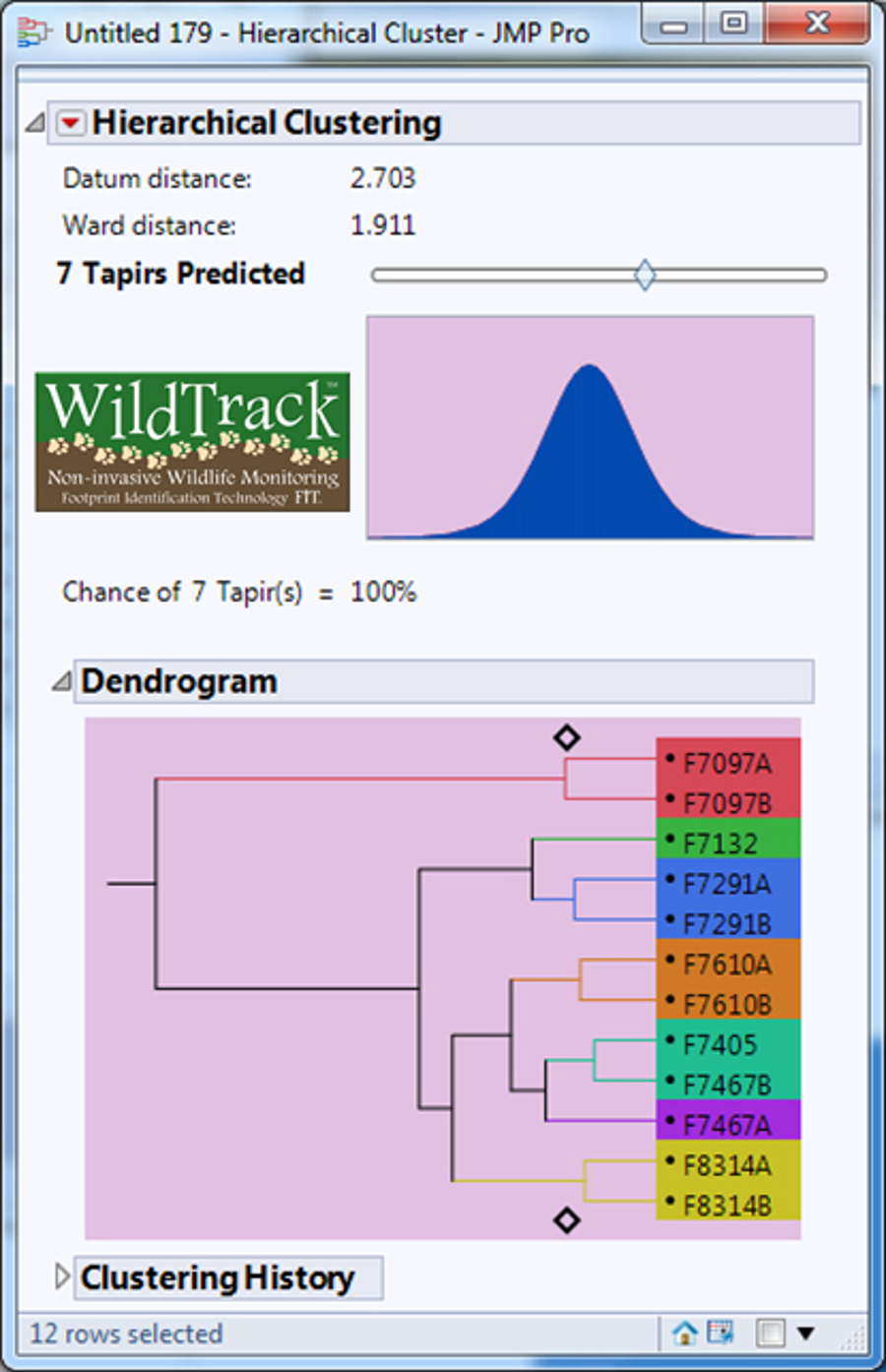

Supplement: Supplemental Information 5 — Each letter represents a location; a number represents a trail; a number and letter, a sub-trail, and the colors represent one individual. [file peerj-06-4591-s005.png]
